# Prediction of long-term major adverse cardiac events after myocardial infarction: value of combination of inflammatory biomarkers and GRACE score

**DOI:** 10.3389/fcvm.2025.1591578

**Published:** 2025-07-10

**Authors:** Dan Tian, Nianxi Yu, Tianxiao Mao, Yanli Li, Ruiyan Liu, Ye Xu, Dong Huang, Qianzhou Lv, Kunming Pan

**Affiliations:** ^1^ Department of Pharmacy, Zhongshan Hospital Fudan University, Shanghai, China; ^2^ Department of Pharmacy, Second Affiliated Hospital of Naval Medical University, Shanghai, China; ^3^ Department of Nursing, Zhongshan Hospital Fudan University, Shangha, China; ^4^ Department of Cardiology, Zhongshan Hospital Fudan University, Shanghai, China; ^5^ Department of Pharmacy, Zhongshan Hospital, Fudan University (Xiamen Branch), Xiamen, Fujian, China

**Keywords:** GRACE score, soluble interleukin-2 receptor, interleukin-8, major adverse cardiovascular events, myocardial infarction

## Abstract

**Objective:**

This study aims to investigate the prognostic value of integrating inflammatory biomarkers with the established Global Registry of Acute Coronary Events (GRACE) risk score for predicting clinical outcomes in patients with myocardial infarction (MI).

**Methods:**

This prospective, single-center study enrolled adult MI patients admitted to the coronary care unit at Zhongshan Hospital, Fudan University. Blood samples were collected to measure inflammatory markers (IL-1β, sIL-2R, IL-6, IL-8) and myocardial biomarkers. The Gensini score and GRACE score were calculated for each patient. The primary endpoint was the post-MI occurrence of a composite of major adverse cardiovascular events (MACE), including cardiovascular death, non-fatal MI, and non-fatal ischemic stroke. Predictive performance of biomarkers was evaluated using Kaplan–Meier survival curves, Cox regression analysis, and receiver operating characteristic (ROC) curves.

**Results:**

A total of 724 patients (median age 64 years, 85.0% male) were included with a median follow-up of 1.7 years. During follow-up, 81 patients (11.1%) experienced MACE, including 45 cardiovascular deaths, 23 MIs, and 13 strokes. Multivariate Cox regression analysis revealed that sIL-2R and IL-8 were independent predictors of MACE. Elevated levels of sIL-2R (HR = 9.123, 95% CI: 5.883–14.147, *p* < 0.001) and IL-8 (HR = 4.443, 95% CI: 2.769–7.131, *p* < 0.001) were significantly associated with an increased risk of MACE. After adjustment for cardiovascular risk factors, elevated sIL-2R (adjusted HR: 3.761, 95% CI: 2.269–6.233, *p* < 0.001) and IL-8 (adjusted HR: 2.294, 95% CI: 1.375–3.825, *p* = 0.001) levels remained significantly associated with an increased risk of MACE. The combination of sIL-2R, IL-8, and GRACE score displayed effective predictive performance for long-term MACE, as evidenced by ROC curve analysis (AUC = 0.824, 95% CI: 0.775–0.873, *p* < 0.001).

**Conclusion:**

Elevated levels of sIL-2R and IL-8 independently predict increased risk of MACE in MI patients. Integrating biomarkers such as sIL-2R and IL-8 with the GRACE score can significantly improve predictive performance, offering a robust approach for risk stratification in MI patients.

## 1 Introduction

Cardiovascular diseases (CVDs) remain the leading cause of premature morbidity and mortality worldwide, accounting for over 40% of all deaths in China (1). A significant subset of these deaths is attributed to severe acute myocardial infarction (AMI) (2). The burden of myocardial infarction (MI) is rising rapidly as China faces an aging population and increasing metabolic risk factors (3).

Despite optimal therapy and secondary prevention measures targeting low-density lipoprotein cholesterol (LDL-C), blood pressure, and glycemia, residual risks of recurrent major adverse cardiovascular events (MACE) post-MI persist. Extensive evidence supports the role of inflammation in the progression of atherosclerosis, formation of unstable plaques, and plaque erosion and rupture (4, 5).

Several inflammatory biomarkers have gained prominence for their role in predicting MACE, such as high-sensitivity CRP (hs-CRP), Interleukin-6 (IL-6), and IL-1β (6, 7). Interleukins (ILs) are a group of proteins secreted by various cells, such as monocytes, macrophages, endothelial cells, and fibroblasts, in response to proinflammatory stimuli (8). Numerous studies have investigated the relationship between ILs and MI (6). In mouse models of MI, IL-1α is released by dying cardiomyocytes (9), and IL-1β synthesis is significantly upregulated post-infarction (10). IL-6 is assumed to be produced by cardiomyocytes during ischemia and reperfusion. In recent studies, IL-6R-targeting monoclonal antibodies improved myocardial salvage in ST-Segment Elevation Myocardial Infarction (STEMI) patients, though adverse events were similar between the treatment and placebo groups (11). Elevated baseline IL-8 levels have been associated with long-term all-cause mortality in male post-MI patients, but there are few reports on other cardiovascular outcomes (12). Moreover, although sIL-2R is recognized for its role in immune response and inflammation, studies directly linking it to MI prognosis in humans remain relatively limited (13).

Despite their predictive value, inflammatory biomarkers face challenges in clinical application. For instance, their specificity is limited, as levels can be influenced by infections, autoimmune diseases, or other non-cardiovascular conditions. Thus, integrating these biomarkers with traditional risk scores and clinical parameters to develop more accurate predictive models remains a key research priority. To bridge this gap, the present study utilizes the Global Registry of Acute Coronary Events (GRACE) score and incorporates interleukin-based biomarkers to predict long-term adverse cardiovascular events in a large cohort of MI patients. This approach is contextualized within a framework of well-established covariates associated with poor clinical outcomes, including congestive heart failure, hypertension, impaired renal function, advanced age, and validated myocardial markers.

## 2 Materials and methods

### 2.1 Study population

**Figure 1:**
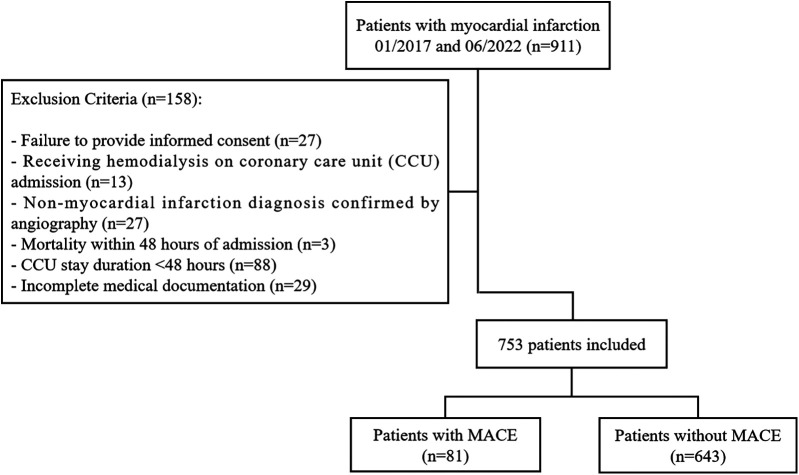
Flowchart of included and excluded patients.

Patients diagnosed with MI and admitted to the coronary care unit (CCU) of Zhongshan Hospital, Fudan University, between January 2017 and June 2022 were prospectively enrolled (14) (Figure 1). Treatment and management were following guideline recommendations (15, 16). Patients were followed up for a minimum of one year with regular evaluations. Exclusion criteria included incomplete follow-up, inability to provide informed consent, unavailable clinical data, and coronary angiography findings inconsistent with MI diagnosis. The study protocol was approved by the Ethics Committee of Zhongshan Hospital, Fudan University (approval number: B2022-375R), and all participants provided written informed consent in accordance with the Declaration of Helsinki.

### 2.2 Biomarker measurements

Blood samples were obtained after 12 h of overnight fasting on the second day of admission to the CCU. All biomarkers were analyzed at the Department of Laboratory Medicine, Zhongshan Hospital, Fudan University. Samples were aliquoted and stored at −70°C until analysis. Interleukin levels (IL-1β, sIL-2R, IL-6, and IL-8) were measured using chemiluminescence on the IMMULITE 1000 automated chemiluminescence immune analyzer (Siemens, Germany), with predefined detection limits. Cardiac troponin T (cTnT) and N-terminal pro-brain natriuretic peptide (NT-proBNP) levels were measured using electrochemiluminescence on the Cobas e 801 (Roche Diagnostics, Germany), while hs-CRP levels were measured using immunoturbidimetry on the Cobas c 702 (Roche Diagnostics, Germany). Routine biochemical analyses were performed using standard laboratory methods. Values below detection limits were recorded as the lowest detectable value.

### 2.3 Follow-up and clinical outcomes

Patients were followed up post-discharge through outpatient visits or telephone interviews at 1, 2, 3, 6, 9, and 12 months, and quarterly thereafter. The follow-up duration ranged from 1 to 7.6 years, with a median of 1.7 years, calculated using the median follow-up time among surviving patients at the study's conclusion.

The primary endpoint was a composite of MACE, defined as a composite of cardiovascular death, non-fatal myocardial infarction, and non-fatal ischemic stroke. Deaths of unknown cause were also classified as MACE.

### 2.4 Calculation of risk scores

The severity of coronary artery stenosis was evaluated using the Gensini scoring system, which assigns a weighted score based on the degree of luminal narrowing: <25% = 1 point, 25%–50% = 2 points, 51%–75% = 4 points, 76%–90% = 8 points, 91%–99% = 16 points, and total occlusion = 32 points. These scores were further adjusted by multiplying with segment-specific coefficients, such as the left main trunk (×5), proximal left anterior descending artery (×2.5), and other segments with coefficients ranging from 0.5 to 2.5, as described in established protocols (17).

The GRACE score, a validated tool for predicting in-hospital and long-term mortality and recurrent MI risk (18), was calculated for each patient upon admission using an online calculator (http://www.outcomes-umassmed.org/grace). The GRACE score incorporates eight clinical variables: age, heart rate, systolic blood pressure, serum creatinine, Killip class, cardiac arrest, presence of ST-segment deviation, and elevated cardiac enzymes or markers.

### 2.5 Statistical analysis

Statistical analyses were conducted using SPSS (version 26.0, IBM Inc., Armonk, NY, USA) and R (version 4.2.1; R Core Team). The normality of continuous variables was evaluated using the Kolmogorov–Smirnov test. Continuous variables were summarized as mean ± standard deviation or median (interquartile range, IQR) and compared using the independent samples t-test or Mann–Whitney U test, as appropriate. Categorical variables were presented as frequencies (percentages) and analyzed using the chi-square test or Fisher's exact test.

Univariate Cox regression analysis was used to explore the relationships between ILs and clinical variables and outcomes. Variables with *p* < 0.1 in univariate analysis, along with clinically relevant factors, were incorporated into multivariate Cox regression models using a stepwise backward elimination approach. Renal insufficiency was defined as estimated glomerular filtration rate (eGFR) < 60 ml/min, calculated using the Chronic Kidney Disease Epidemiology Collaboration (CKD-EPI) equation.

Biomarker levels were stratified using optimal cut-off values determined by receiver operating characteristic (ROC) curve analysis. Kaplan–Meier curves were plotted based on follow-up time (years), and the cumulative survival rates between high and low IL level groups were estimated using the log-rank test. The predictive performance of IL-based models, both independently and in combination with established risk scores, was quantified using area under the ROC curve (AUC) analysis. All data were analyzed using two-sided tests, with *p* < 0.05 considered statistically significant.

## 3 Results

### 3.1 Patient characteristics according to MACE

A cohort of 724 patients (85% male, with a median age of 64 years) was followed for a median duration of 1.7 years. During follow-up, 81 patients (11.1%) reached the primary endpoint, comprising 45 cardiovascular deaths, 23 MIs, and 13 strokes. Patients with MACE were older and had a higher prevalence of cardiovascular risk factors, including HR > 100 b.p.m., diabetes, coronary artery disease, heart failure, history of percutaneous coronary intervention (PCI) or coronary artery bypass grafting (CABG), renal insufficiency, and anemia (Table 1). These patients had significantly higher GRACE scores and elevated levels of cTnT, hs-CRP, NT-proBNP, sIL-2R, IL-6, and IL-8. However, there were no significant differences in creatine kinase (CK), CK-MB, and Gensini scores between groups. Medication analysis revealed that patients without MACE were more frequently prescribed aspirin, ticagrelor, beta-blockers, angiotensin-converting enzyme inhibitor (ACEI) or angiotensin receptor blocker (ARB), and statins compared to those with MACE.

**Table 1:** Baseline characteristics of study participants stratified by major adverse cardiovascular events (MACE) status during 1.7-year follow-up.

Variables	All patients *N* = 724	MACE *N* = 81	No-MACE *N* = 643	*P* value
Demographic information				
Age, years	64.1 (15.1)	72.0 (15.8)	63.4 (15.2)	<0.001
Male	606 (83.9)	63 (77.8)	543 (84.7)	0.109
Body mass index, kg/m^2^	24.5 (4.2)	23.5 (3.6)	24.6 (4.3)	0.260
Cardiovascular risk factor				
STEMI (vs. NSTEMI)	430 (59.4)	46 (56.8)	384 (59.7)	0.613
Heart rate (>100 b.p.m.)	72 (10.0)	20 (25.3)	52 (8.1)	<0.001
SBP (<100 mmHg)	73 (10.1)	12 (15.2)	61 (9.5)	0.114
Current smoker	315 (43.5)	28 (34.6)	287 (44.6)	0.085
Diabetes mellitus	196 (27.1)	33 (40.7)	163 (25.3)	0.003
Hypertension	417 (57.6)	52 (64.2)	365 (56.8)	0.202
Hypercholesterolemia	55 (7.6)	6 (7.4)	49 (7.6)	0.946
Coronary artery disease	223 (30.8)	39 (48.1)	184 (28.6)	<0.001
Arrhythmia	52 (72.0)	9 (11.1)	43 (6.7)	0.146
Heart failure	36 (5.0)	12 (14.8)	24 (3.7)	<0.001
History of PCI or CABG	80 (11.0)	15 (18.5)	65 (10.1)	0.023
History of stroke	37 (5.1)	6 (7.4)	31 (4.8)	0.466
Renal insufficiency (eGFR <60 ml/min)	153 (21.2)	45 (55.6)	108 (16.8)	<0.001
Anemia	237 (32.7)	43 (53.1)	194 (30.2)	<0.001
GRACE score	104.0 (38.0)	128.0 (37.0)	99.0 (37.0)	<0.001
Gensini score	59.0 (45.0)	68.0 (55.0)	58.0 (44.0)	0.205
Laboratory tests				
HbAlc, %	6.1 (1.5)	6.4 (2.2)	6.0 (1.4)	0.023
eGFR, ml/min	80.0 (29.0)	57.0 (42.6)	82.0 (26.0)	<0.001
TC, mmol/L	4.5 (1.4)	4.0 (1.7)	4.5 (1.4)	0.002
LDL-C, mmol/L	2.6 ± 1.0	2.3 ± 1.0	2.6 ± 1.0	0.004
HDL-C, mmol/L	1.0 (0.3)	1.0 (0.3)	1.0 (0.3)	0.928
TG, mmol/L	1.4 (1.1)	1.3 (0.9)	1.4 (1.1)	0.909
AST, U/L	65.0 (98.0)	74.0 (151.0)	63.0 (99.0)	0.090
ALT, U/L	33.0 (27.0)	34.0 (43.0)	33.0 (26.0)	0.459
PLT, × 10^9^/L	205.0 (71.0)	182.0 (77.0)	207.0 (69.0)	0.001
WBC, × 10^9^/L	8.8 (3.7)	9.8 (4.8)	8.7 (3.5)	0.001
Albumin, g/L	40.0 (5.0)	37.0 (7.0)	40.0 (5.0)	<0.001
cTnT, ng/ml	2.0 (3.2)	2.5 (4.9)	1.9 (3.1)	0.021
hs-CRP, mg/dl	13.1 (33.1)	28.6 (60.8)	12.2 (31.0)	0.001
NT-pro BNP, pg/ml	974.0 (1,652.0)	2,976.0 (5,225.0)	856.0 (1,430.0)	<0.001
CK, U/L	420.0 (820.0)	505.0 (728.0)	413.5 (824.0)	0.780
CK-MB, U/L	36.0 (53.0)	42.0 (49.0)	35.0 (53.0)	0.349
IL-1β, pg/ml	5.0 (1.4)	5.0 (1.9)	5.0 (1.4)	0.843
sIL-2R, U/ml	408.0 (206.0)	624.0 (509.0)	396.0 (181.0)	<0.001
IL-6, pg/ml	13.1 (18.2)	28.5 (41.7)	12.0 (15.9)	<0.001
IL-8, pg/ml	15.0 (17.0)	26.0 (31.5)	14.0 (15.0)	<0.001
Medications after discharge				
Antiplatelet therapy				<0.001
Monotherapy with antiplatelets	34 (4.7)	4 (4.9)	30 (4.7)	
Dual antiplatelet therapy	677 (93.5)	69 (85.2)	608 (94.6)	
β-blockers	571 (78.9)	52 (64.2)	519 (80.7)	0.001
ACEI/ARB	606 (83.7)	55 (67.9)	551 (85.7)	<0.001
Statins	682 (94.2)	70 (86.4)	612 (95.2)	0.001
Loop diuretics	193 (26.7)	31 (38.3)	162 (25.2)	0.012
Spironolactone	178 (24.6)	22 (27.2)	156 (24.3)	0.568
Nitrate drugs	200 (27.6)	28 (34.6)	172 (26.7)	0.138
Calcium channel blockers	92 (12.7)	12 (14.8)	80 (12.4)	0.546
Anticoagulants	44 (6.1)	7 (8.6)	37 (5.8)	0.305

Values are presented as mean ± standard deviation, median (interquartile range) or % (*n*). MACE, major adverse cardiovascular events; STEMI, ST-elevation myocardial infarction; NSTEMI, non-ST-elevation myocardial infarction; SBP, systolic blood pressure; PCI, percutaneous coronary intervention; CABG, coronary artery bypass grafting; eGFR, estimated glomerular filtration rate; GRACE, Global Registry of Acute Coronary Events; TC, total cholesterol; LDL-C, low-density lipoprotein cholesterol; HDL-C, high-density lipoprotein cholesterol; TG, triglycerides; AST, aspartate aminotransferase; ALT, alanine aminotransferase; PLT, platelet count; WBC, white blood cell count; cTnT, Cardiac troponin T; hs-CRP, high sensitivity C-reactive protein; NT-proBNP, N-terminal pro-B-type natriuretic peptide; CK, creatine kinase; IL, interleukin; sIL-2R, soluble IL-2 receptor; ACEI, angiotensin-converting enzyme inhibitor; ARB, angiotensin receptor blocker.

### 3.2 Determination of risk factors for MACE

Univariate Cox analysis identified several parameters associated with increased risk of MACE, including age, HR > 100 b.p.m., diabetes, coronary artery disease, heart failure, history of PCI or CABG, renal insufficiency, anemia, GRACE score, lower levels of serum total cholesterol (TC), LDL-C, and platelet (PLT), as well as elevated levels of aspartate aminotransferase (AST), alanine aminotransferase (ALT), white blood cell (WBC), cTnT, hs-CRP, NT-proBNP, CK, CK-MB, sIL-2R, IL-6, and IL-8 (Supplementary Table S1). Subsequent multivariate Cox regression analyses using backward stepwise regression models demonstrated that diabetes mellitus, higher GRACE scores, and indicators of inflammatory-immune activation (sIL-2R, IL-8, and WBC) were significantly associated with an elevated risk of MACE (Supplementary Table S2).

### 3.3 sIL-2R and IL-8 as predictive biomarkers for MACE

**Figure 2:**
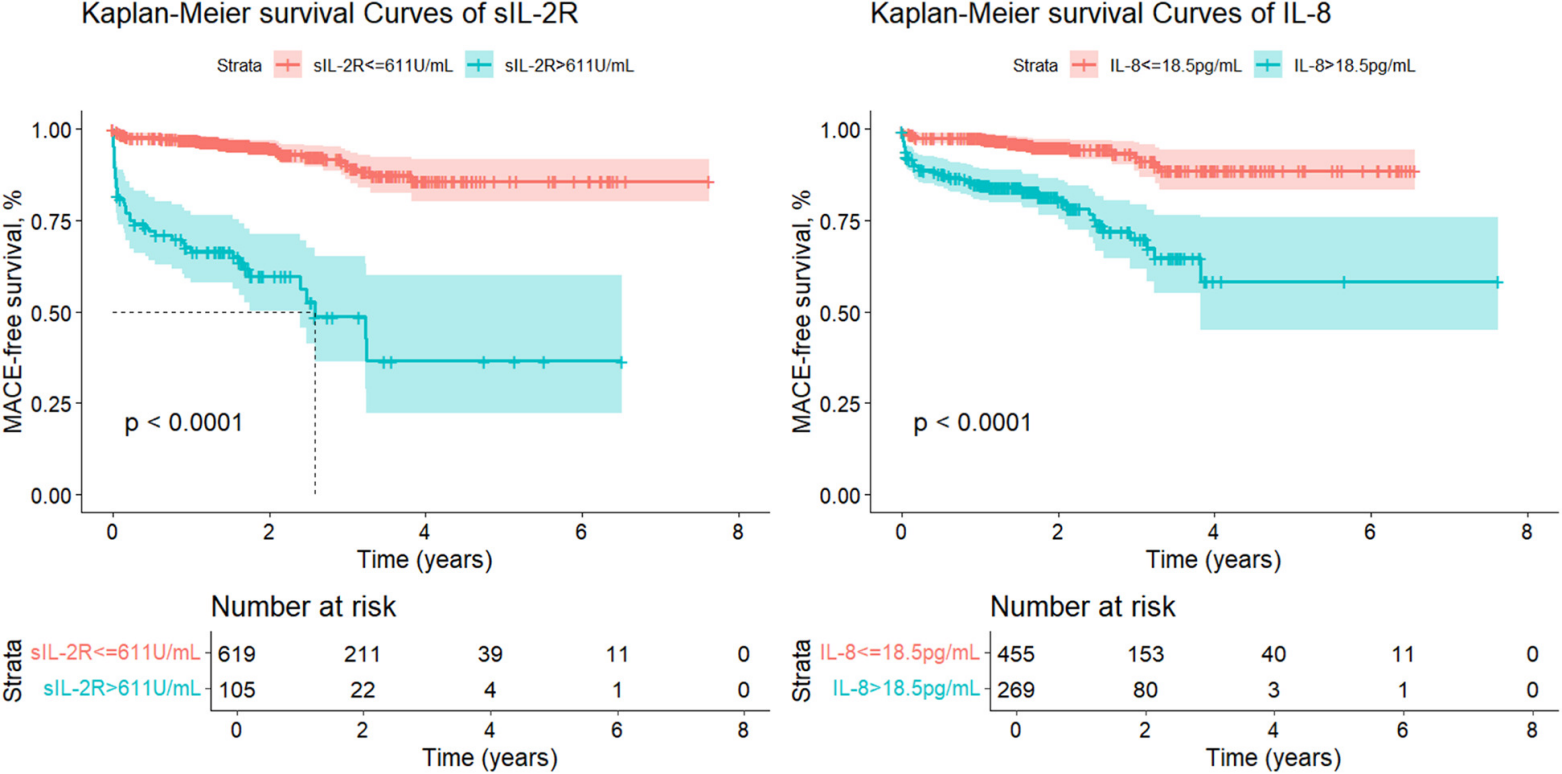
Kaplan–Meier curves for MACE according to sIL-2R and IL-8. MACE, major adverse cardiovascular events; sIL-2R, soluble interleukin-2 receptor; IL-8, interleukin-8.

ROC analysis demonstrated that both sIL-2R and IL-8 effectively discriminated between patients with and without MACE. The optimal cut-off values were 611 U/ml for sIL-2R (sensitivity 53.1%; specificity 90.4%) and 18.5 pg/ml for IL-8 (sensitivity 69.1%; specificity 66.9%). Patients were further grouped based on the cut-off value of each variable. As shown in Figure 2, patients were stratified into high-level and low-level groups for sIL-2R and IL-8. The analysis demonstrated that both high-level sIL-2R and high-level IL-8 were significantly associated with inferior MACE-free survival compared to their respective low-level groups (log-rank test, *p* < 0.0001 for both). Specifically, patients with elevated levels of sIL-2R had a significantly higher risk of MACE during follow-up (HR = 9.123, 95% CI: 5.883–14.147, *p* < 0.001), which remained significant after adjusting for cardiovascular risk factors identified in this study (adjusted HR = 3.761, 95% CI: 2.269–6.233, *p* < 0.001) (Supplementary Table S2). Similarly, elevated IL-8 levels were associated with a higher risk of MACE (HR = 4.443, 95% CI: 2.769–7.131, *p* < 0.001), with the association persisting after adjustment for relevant cardiovascular risk factors (adjusted HR = 2.294, 95% CI: 1.375–3.825, *p* = 0.001) (Table 2).

**Table 2:** Cox regression analysis of high IL levels and the risk of MACE.

Models	Variables	HR (95% CI)	*P* value
Univariate model	High sIL-2R level	9.123 (5.883–14.147)	<0.001
	High IL-8 level	4.443 (2.769–7.131)	<0.001
Model 1	High sIL-2R level	3.761 (2.269–6.233)	<0.001
	High IL-8 level	2.294 (1.375–3.825)	0.001
	GRACE score	1.026 (1.018–1.035)	<0.001
	PLT, × 10^9^/L	0.996 (0.993–1.000)	0.045
	WBC, × 10^9^/L	1.009 (1.001–1.017)	0.031
	NT-pro BNP, pg/ml	1.000 (1.000–1.000)	0.063
	Diabetes mellitus	1.497 (0.950–2.358)	0.082

Patients were further grouped based on the cut-off value of IL levels. Values exceeding the optimal cut-off value are defined as the high IL level group. Model 1 was constructed through backward stepwise regression modeling, incorporating dichotomized sIL-2R, IL-8, and cardiovascular risk factors identified by multivariate Cox regression analysis, specifically including diabetes mellitus, GRACE score, LDL-C, PLT, WBC, and NT-pro BNP. MACE, major adverse cardiovascular events; sIL-2R, soluble interleukin-2 receptor; IL-8, interleukin-8; GRACE, Global Registry of Acute Coronary Events; LDL-C, low-density lipoprotein cholesterol; PLT, platelet count; WBC, white blood cell count; NT-pro BNP, N-terminal pro-B-type natriuretic peptide.

### 3.4 Superior predictive performance of combined sIL-2R, IL-8, and GRACE model

**Figure 3:**
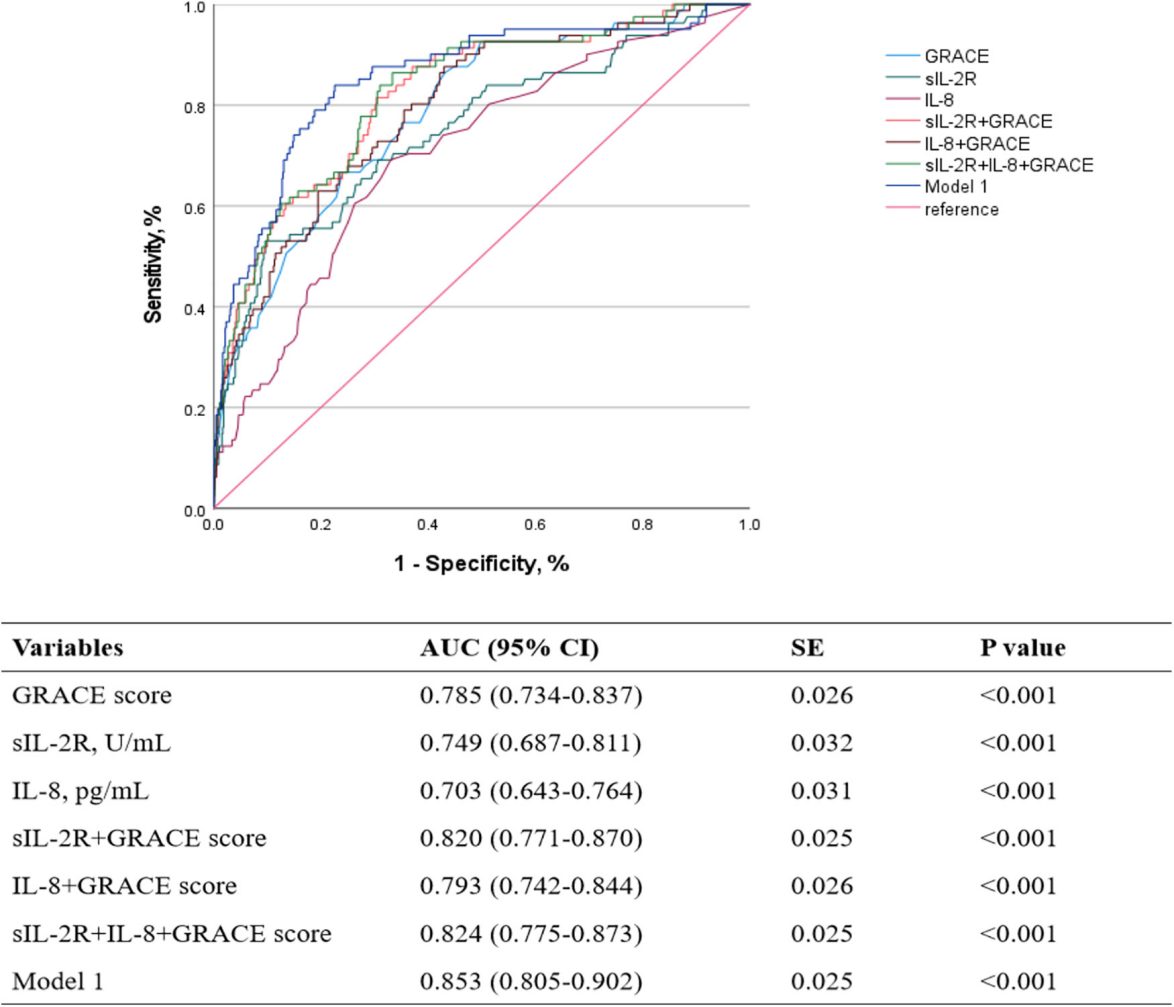
ROC curves for the predictive model discriminating MACE based on different indicators. ROC, receiver operating characteristic; MACE, major adverse cardiovascular events; AUC, area under curve; SE, standard error; sIL-2R, soluble interleukin-2 receptor; IL-8, interleukin-8; GRACE, Global Registry of Acute Coronary Events.

The GRACE score demonstrated the highest individual predictive value, with an AUC of 0.785 (95% CI: 0.734–0.837, *p* < 0.001). Among the biomarkers, sIL-2R and IL-8 showed significant predictive performance, with AUCs of 0.749 (95% CI: 0.687–0.811, *p* < 0.001) and 0.703 (95% CI: 0.643–0.764, *p* < 0.001), respectively. The combination of multiple indicators is significantly superior to single-variable prediction. The synergistic effect of inflammatory factors (sIL-2R/IL-8) and GRACE score can improve the prediction accuracy. Notably, the combination of sIL-2R, IL-8, and GRACE score displayed effective predictive performance for long-term MACE, as evidenced by ROC curve analysis (AUC = 0.824, 95% CI: 0.775–0.873, *p* < 0.001). Model 1 was constructed via backward stepwise regression modeling using dichotomized sIL-2R, IL-8, and cardiovascular risk factors identified by multivariate Cox regression. Model 1 was identified as the optimal combination, with an AUC of 0.853 (95% CI: 0.805–0.902, *p* < 0.001) (Figure 3).

## 4 Discussion

Our study indicated that higher levels of sIL-2R (>611 U/ml) and IL-8 (>18.5 pg/ml) were significantly associated with increased long-term post-MI risk of MACE, even after adjusting for cardiovascular risk factors. ROC analysis showed that sIL-2R had the best predictive performance for MACE, and the combination of sIL-2R, IL-8, and GRACE score was the most predictive.

Inflammation is a pivotal factor in the progression of atherosclerosis, plaque formation, and rupture, particularly in patients with MI (4, 19). The process is driven by cytokines such as IL-1, IL-6, and IL-8, which activate monocyte-macrophages, leading to the release of additional cytokines, including IL-2 (8, 20–22). IL-2 promotes T cell activation, leading to the proteolytic cleavage of the IL-2 receptor α-chain (CD25) and the release of sIL-2R, a 45-kDa glycoprotein that serves as a marker of immune activation (23–25). Elevated sIL-2R levels reflect heightened immune activation, contributing to endothelial dysfunction, atherosclerotic plaque instability, and adverse cardiovascular outcomes in MI patients (13). Our previous work demonstrated that elevated baseline sIL-2R levels were associated with poor outcomes in MI patients (26). A recent study further identified sIL-2R as an independent predictor of acute kidney injury and in-hospital all-cause mortality in MI patients, with ROC cut-off values of 423 U/ml and 615 U/ml, respectively (27). In this study, the sIL-2R cut-off value for predicting MACE was 611 U/ml, consistent with previous findings, supporting the external validity of our results.

IL-8 is also implicated in atherosclerotic plaque destabilization and thrombosis (28), and inhibits cholesterol efflux, contributing to lipid accumulation and the progression of atherosclerosis (29). IL-8 concentrations are elevated in patients with ACS compared to those with chronic stable angina (30). Also, elevated baseline IL-8 levels are linked to an increased risk of long-term mortality, independent of various clinical, laboratory, and angiographic factors (12). Although the precise mechanisms remain unclear, IL-8's involvement in atherogenesis, plaque destabilization, and thrombosis likely underlies this association. Our study not only confirms the link between elevated IL-8 levels and post-MI mortality but also highlights its predictive value for composite endpoint events, underscoring its potential as a prognostic biomarker in MI.

Clinicians traditionally rely on risk scores and biomarkers to predict MACE (31). Widely used risk scores such as the GRACE score and Thrombolysis in Myocardial Infarction (TIMI) score are based on clinical parameters (32, 33). However, the TIMI score exhibits lower accuracy than the GRACE score in predicting long-term outcomes. This study found that the GRACE score had the highest individual predictive performance (AUC = 0.785), consistent with its established use in acute coronary syndrome (ACS) patients (18). However, these scores fail to effectively incorporate underlying pathophysiological mechanisms such as inflammation, and their predictive accuracy is lower in patients with complex comorbidities or atypical presentations in MI patients. In our study, sIL-2R and IL-8 demonstrated significant predictive value. By integrating biomarkers like sIL-2R and IL-8 with the GRACE score, we found that the predictive performance was significantly enhanced. This integration provides a robust method for risk stratification in myocardial infarction (MI) patients. Traditional biomarkers, such as troponin T/I and BNP/NT-proBNP, primarily reflect myocardial damage or cardiac stress, however, they fail to provide a comprehensive assessment of systemic risk, particularly the role of inflammation in cardiovascular events (34).

Platelets play a crucial role in thrombosis, making antiplatelet therapy essential for post-PCI MI patients (35). However, the association between baseline platelet levels and long-term MACE remains controversial. Yadav M et al. analyzed 10,603 Non-ST-segment elevation myocardial infarction (NSTEMI) or STEMI patients from the ACUITY and HORIZONS-AMI trials, finding that baseline thrombocytopenia significantly increased MACE rates (20.8% vs. 15.6%; *p* = 0.0002). Multivariate analysis confirmed thrombocytopenia as an independent MACE predictor (HR = 1.39, *p* = 0.009) (36). Conversely, Liu R et al.'s study of 16,957 STEMI patients from the CAMI registry showed higher MACE rates in thrombocytopenic patients (23.6% vs. 13.9%, *p* < 0.001), but this was not independent of other factors (HR = 1.18, *p* = 0.132) (37). These discrepancies may result from differences in controlling confounders, such as hemoglobin. Additionally, Brodsky MA et al. reported a high MACE incidence (28.6%) in immune-mediated thrombocytopenic purpura (iTTP) survivors over 7.6 years (38). Our study confirms a significant, independent link between baseline thrombocytopenia and long-term MACE, emphasizing the importance of monitoring platelet levels in clinical practice.

This study has several limitations. First, the cross-sectional design of biomarker assessment may introduce bias, as ILs' levels could fluctuate during disease progression, particularly before and after PCI, and variations in detection time points may affect result interpretation and limit causal inference. Second, the single-center design and absence of an external validation cohort restrict the generalizability of our findings. Future large-scale, multicenter prospective studies with extended follow-up periods are warranted to provide more robust evidence and validate these results.

## 5 Conclusion

In conclusion, our findings indicate that elevated sIL-2R and IL-8 levels are independent predictors of long-term MACE in MI patients. The integration of biomarkers such as sIL-2R and IL-8 with the GRACE score can significantly improve predictive performance, offering a robust approach for risk stratification in MI patients.

## Data Availability

The original contributions presented in the study are included in the article/Supplementary Material, further inquiries can be directed to the corresponding authors.
