# Supplementary material for: Prediction of long-term major adverse cardiac events after myocardial infarction: value of combination of inflammatory biomarkers and GRACE score
Source: Front Cardiovasc Med. 2025 Jul 10;12:1591578. doi: 10.3389/fcvm.2025.1591578 (PMC12287059; doi:10.3389/fcvm.2025.1591578)
Supplement: Supplementary file 1 [file Datasheet1.pdf]

SUPPLEMENTARY TABLES

Table S1. Univariate Cox regression analysis of patients with and without MACE during long-term follow-up

Variables	All patients N=724	MACE N=81	No-MACE N=643	HR (95% CI)	P value
Demographic Information					
Age, years	64.1(15.1)	72.0(15.8)	63.4(15.2)	1.082 (1.058-1.106)	<0.001
Male	606(83.9)	63(77.8)	543(84.7)	0.574 (0.339-0.972)	0.039
Body mass index, kg/m^2	24.5(4.2)	23.5(3.6)	24.6(4.3)	0.960 (0.873-1.056)	0.405
Cardiovascular Risk Factors					
STEMI (vs. NSTEMI)	430(59.4)	46(56.8)	384(59.7)	0.893 (0.576-1.387)	0.616
Heart rate >100 b.p.m.	72(10.0)	20(25.3)	52(8.1)	3.744 (2.253-6.223)	<0.001
SBP <100 mmHg	73(10.1)	12(15.2)	61(9.5)	1.823 (0.986-3.370)	0.056
Current smoker	315(43.5)	28(34.6)	287(44.6)	0.645 (0.408-1.020)	0.061
Diabetes mellitus	196(27.1)	33(40.7)	163(25.3)	2.015 (1.293-3.139)	0.002
Hypertension	417(57.6)	52(64.2)	365(56.8)	1.320 (0.838-2.079)	0.231
Hypercholesterolemia	55(7.6)	6(7.4)	49(7.6)	0.981 (0.427-2.254)	0.964
Coronary artery disease	223(30.8)	39(48.1)	184(28.6)	1.875 (1.209-2.907)	0.005
Arrhythmia	52(72.0)	9(11.1)	43(6.7)	1.669 (0.834-3.343)	0.148
Heart failure	36(5.0)	12(14.8)	24(3.7)	3.698 (2.001-6.832)	<0.001
History of PCI or CABG	80(11.0)	15(18.5)	65(10.1)	2.109 (1.202-3.699)	0.009
History of stroke	37(5.1)	6(7.4)	31(4.8)	1.778 (0.772-4.093)	0.176
Renal insufficiency	153(21.2)	45(55.6)	108(16.8)	5.579 (3.595-8.657)	<0.001
Anemia	237(32.7)	43(53.1)	194(30.2)	2.688 (1.736-4.163)	<0.001
GRACE score	104.0(38.0)	128.0(37.0)	99.0(37.0)	1.041 (1.032-1.049)	<0.001
Gensini score	59.0(45.0)	68.0(55.0)	58.0(44.0)	1.003 (0.997-1.008)	0.335
Laboratory tests					
TC, mmol/L	4.5(1.4)	4.0(1.7)	4.5(1.4)	0.740 (0.590-0.928)	0.009
LDL-C, mmol/L	2.6±1.0	2.3±1.0	2.6±1.0	0.682 (0.531-0.876)	0.003
HDL-C, mmol/L	1.0(0.3)	1.0(0.3)	1.0(0.3)	0.801 (0.336-1.908)	0.616
TG, mmol/L	1.4(1.1)	1.3(0.9)	1.4(1.1)	0.875 (0.687-1.115)	0.281
AST, U/L	65.0(98.0)	74.0(151.0)	63.0(99.0)	1.0005 (1.0003-1.0006)	<0.001
ALT, U/L	33.0(27.0)	34.0(43.0)	33.0(26.0)	1.0008 (1.0006-1.0010)	<0.001
PLT, ×10^9/L	205.0(71.0)	182.0(77.0)	207.0(69.0)	0.993 (0.989-0.997)	0.001
WBC, ×10^9/L	8.8(3.7)	9.8(4.8)	8.7(3.5)	1.014 (1.008-1.021)	<0.001
Albumin ,g/L	40.0(5.0)	37.0(7.0)	40.0(5.0)	0.833 (0.794-0.875)	<0.001
cTnT, ng/mL	2.0(3.2)	2.5(4.9)	1.9(3.1)	1.126 (1.044-1.215)	0.002
hs-CRP, mg/dL	13.1(33.1)	28.6(60.8)	12.2(31.0)	1.008 (1.005-1.011)	<0.001
NT-proBNP, pg/mL	974.0(1652.0)	2976.0(5225.0)	856.0(1430.0)	1.00010 (1.00008-1.00012)	<0.001
CK, U/L	420.0(820.0)	505.0(728.0)	413.5(824.0)	1.0002 (1.0001-1.0004)	<0.001
CK-MB, U/L	36.0(53.0)	42.0(49.0)	35.0(53.0)	1.003 (1.002-1.005)	<0.001
IL-1β, pg/mL	5.0(1.4)	5.0(1.9)	5.0(1.4)	1.006 (0.99-1.023)	0.469
sIL-2R, U/mL	408.0(206.0)	624.0(509.0)	396.0(181.0)	1.0010 (1.0009-1.0012)	<0.001
IL-6, pg/mL	13.1(18.2)	28.5(41.7)	12.0(15.9)	1.003 (1.002-1.004)	<0.001

IL-8, pg/mL	15.0(17.0)	26.0(31.5)	14.0(15.0)	1.0023 (1.0016-1.0029)	<0.001
Medications after discharge					
Dual antiplatelet therapy	677(93.5)	69(85.2)	608(94.6)	0.326 (0.176-0.602)	<0.001
β-blockers	571(78.9)	52(64.2)	519(80.7)	0.386 (0.245-0.610)	<0.001
ACEI/ARB	606(83.7)	55(67.9)	551(85.7)	0.355 (0.222-0.565)	<0.001
Statins	682(94.2)	70(86.4)	612(95.2)	0.302 (0.160-0.571)	<0.001
Loop diuretics	193(26.7)	31(38.3)	162(25.2)	1.858 (1.186-2.910)	0.007
Spironolactone	178(24.6)	22(27.2)	156(24.3)	1.148 (0.704-1.874)	0.580
Nitrate drugs	200(27.6)	28(34.6)	172(26.7)	1.252 (0.790-1.985)	0.338
Calcium channel blockers	92(12.7)	12(14.8)	80(12.4)	1.194 (0.647-2.205)	0.571
Anticoagulants	44(6.1)	7(8.6)	37(5.8)	1.499 (0.690-3.255)	0.307

Values are presented as mean ± standard deviation, median (interquartile range) or n (%) MACE, major adverse cardiovascular events; HR, hazard ratio; CI, confidence interval; STEMI, ST-elevation myocardial infarction; NSTEMI, non-ST-elevation myocardial infarction; SBP, systolic blood pressure; PCI, percutaneous coronary intervention; CABG, coronary artery bypass grafting; TC, total cholesterol; LDL-C, low-density lipoprotein cholesterol; HDL-C, high-density lipoprotein cholesterol; TG, triglycerides; AST, aspartate aminotransferase; ALT, alanine aminotransferase; PLT, Platelet; WBC, white blood cell; cTnT, Cardiac troponin T; hsCRP, high sensitivity C-reactive protein; NT-proBNP, N-terminal pro-B-type natriuretic peptide; CK, creatine kinase; IL, interleukin; sIL-2R, soluble IL-2 receptor; ACEI, angiotensin-converting enzyme inhibitor; ARB, angiotensin receptor blocker.

Table S2. Multivariate Cox regression analysis of factors associated with MACE during long-term follow-up

Variables	HR (95% CI)	P value
Diabetes mellitus	3.070 (1.248-7.554)	0.015
GRACE score	1.046 (1.029-1.063)	<0.001
LDL-C, mmol/L	0.510 (0.292-0.890)	0.018
PLT, $\times 10^9$/L	0.990 (0.983-0.997)	0.009
WBC, $\times 10^9$/L	1.239 (1.108-1.387)	<0.001
NT-proBNP, pg/mL	1.000 (1.000-1.000)	0.013
sIL-2R, U/mL	1.003 (1.002-1.004)	<0.001
IL-8, pg/mL	1.015 (1.003-1.026)	0.011

Multivariate Cox regression models were constructed using a stepwise backward elimination approach. MACE, major adverse cardiovascular events; HR, hazard ratio; CI, confidence interval; GRACE, Global Registry of Acute Coronary Events; LDL-C, low-density lipoprotein cholesterol; PLT, platelet count; WBC, white blood cell count; NT-proBNP, N-terminal pro-B-type natriuretic peptide; sIL-2R, soluble interleukin-2 receptor; IL-8, interleukin-8.
